# Carotid Intraplaque Neovascularization and Future Vascular Events in Patients With Asymptomatic Carotid Stenosis

**DOI:** 10.3389/fphar.2022.804810

**Published:** 2022-02-22

**Authors:** Liuping Cui, Yingqi Xing, Lijuan Wang, Kangding Liu, Hongxiu Chen, Cong Li, Ying Chen

**Affiliations:** ^1^ Department of Neurology, the First Hospital of Jilin University, Changchun, China; ^2^ Department of Vascular Ultrasonography, Xuanwu Hospital, Capital Medical University, Beijing, China; ^3^ Beijing Diagnostic Center of Vascular Ultrasound, Beijing, China; ^4^ Center of Vascular Ultrasonography, Beijing Institute of Brain Disorders, Collaborative Innovation Center for Brain Disorders, Capital Medical University, Beijing, China

**Keywords:** carotid stenosis, ischemia, neovascularization, ultrasonography, proportional hazards models

## Abstract

**Objective:** Intraplaque neovascularization is a marker of plaque vulnerability and is used to predict the risk of future vascular events in patients with symptomatic carotid stenosis; however, its association with asymptomatic carotid stenosis has not been prospectively evaluated**.** Therefore, this study aimed to explore the association between intraplaque neovascularization assessed using contrast-enhanced ultrasound and the occurrence of future ischemic events in asymptomatic patients diagnosed with carotid stenosis.

**Methods:** We recruited patients with asymptomatic carotid stenosis from our center. Contrast-enhanced ultrasound was performed at baseline. The outcomes were ischemic stroke and cardiovascular events. We plotted Kaplan-Meier survival curves and performed a log-rank test to compare endpoint event probability in patients with and without grade 2 intraplaque neovascularization. Univariate and multivariate Cox proportional hazards models were used to assess predictors of future vascular events.

**Results:** The data of 50 participants were included in the analysis (median follow-up, 43.7 months). Endpoint events occurred in 12 participants (24%). The Kaplan-Meier survival curves showed that patients with grade 2 intraplaque neovascularization had a higher probability of future vascular events than those with grades 0 and 1 (*p* < .05). Grade 2 intraplaque neovascularization (hazard ratio: 4.530, 95% confidence interval, 1.337–15.343, *p* < .05) was an independent predictor of future vascular events in patients with asymptomatic carotid stenosis.

**Conclusion:** Grade 2 intraplaque neovascularization assessed using contrast-enhanced ultrasound independently predicted future ischemic events in patients with asymptomatic carotid stenosis, and contrast-enhanced ultrasound may be an effective screening method to identify high-risk subgroups of patients with asymptomatic carotid stenosis.

## Introduction

Atherosclerosis is one of the leading causes of ischemic cardiovascular and cerebrovascular events. Because it is a systemic disease, the detection of atherosclerotic plaques in one vascular region may suggest a wider pathological condition (Ibanez et al., 2021). Carotid atherosclerotic plaques are visual indicators of systemic atherosclerosis, and vulnerable carotid plaques are closely related to the occurrence of ischemic events (Saba et al., 2019; Zhu et al., 2020). Intraplaque neovascularization (IPN) is a characteristic feature of carotid plaque vulnerability. Lesions with IPN are prone to intraplaque hemorrhage, which is associated with rupture, formation of local thrombi, and distal embolization (Moreno et al., 2004; Xu et al., 2015).

Contrast-enhanced ultrasound (CEUS) is used to examine plaque vulnerability by visualizing intraplaque neovessels in real time. According to studies of symptomatic carotid stenosis patients, IPN assessment using CEUS is independently associated with an increased risk of ischemic stroke after adjusting for the degree of stenosis (Li et al., 2019; Camps-Renom et al., 2020; Song et al., 2020). In addition, it may serve as a clinically useful tool for cardiovascular risk stratification in patients with coronary events (Nakamura et al., 2015; Mantella et al., 2019). Since the risk stratification of patients with asymptomatic carotid stenosis also requires focusing on carotid plaque characteristics, detecting high-risk plaques may help monitor disease progression and help physicians make definitive treatment decisions (Markus et al., 2010; Paraskevas et al., 2014; Baradaran et al., 2019). One recent meta-analysis suggests that patients with asymptomatic carotid stenosis who have high-risk plaques have a higher associated risk of ipsilateral ischemic cerebrovascular events than currently accepted estimates. Specifically, the analysis shows that high-risk plaques were common in patients with asymptomatic carotid stenosis, and IPN was one of the prevalent characteristics of high-risk plaques (Kamtchum-Tatuene et al., 2020). However, our understanding of the role of IPN assessed by CEUS in future ischemic events in patients with asymptomatic carotid stenosis remains limited.

Therefore, in this prospective study, we aimed to explore whether IPN detected by CEUS is associated with the occurrence of future ischemic events in patients with asymptomatic carotid stenosis. We hypothesized that the presence of grade 2 IPN as assessed by CEUS would predict future ischemic events in patients.

## Methods

### Study Population

**FIGURE 1:**
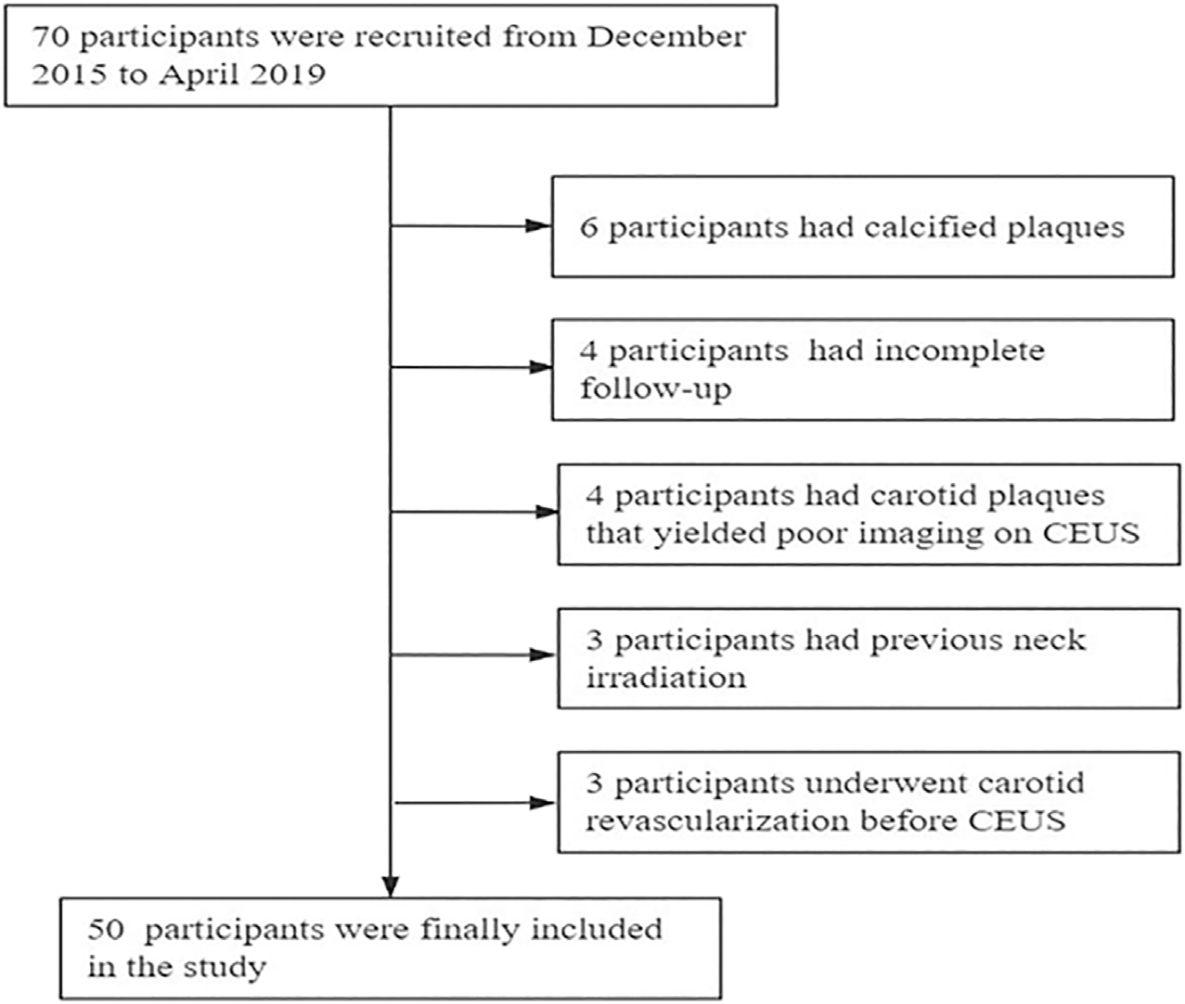
Study flow chart.

This was a prospective observational study. We consecutively recruited 70 participants from the First Hospital of Jilin University between December 2015 and april 2019. Asymptomatic patients with at least one atherosclerotic carotid plaque associated with carotid stenosis were included in the study. Patients were excluded based on the following criteria: 1) underwent carotid revascularization before CEUS; 2) presence of calcified plaque; 3) carotid plaques had poor imaging on CEUS; 4) previous neck irradiation; and 5) incomplete follow-up (Figure 1).

The study was approved by the ethics committee of the First Hospital of Jilin University (No. 2015-285), and the study participants signed an informed consent form.

### Ultrasonography Imaging

Before CEUS imaging was performed, all participants underwent routine carotid ultrasonography. A sonographer with 10 years of experience (CY) examined the bilateral common carotid artery, carotid bifurcation, and internal carotid arteries in the longitudinal and transverse planes. According to the Gray-Weale visual classification system, plaque echogenicity was graded as follows: uniformly hypoechoic (type I), predominantly hypoechoic (type II), predominantly hyperechoic (type III), uniformly hyperechoic (type IV), or extensively calcified (type V) (Gray-Weale et al., 1988). According to the degree of stenosis, as defined by the North American Symptomatic Carotid Endarterectomy Trial, carotid stenosis was divided into mild (< 50%), moderate (50–69%), or severe (≥70%) (von Reutern et al., 2012). When observing the plaques, plaque size, location, echogenicity, and degree of carotid stenosis were recorded. If a participant had multiple plaques, the plaque with the greatest thickness was selected for analysis. Next, the plaques were examined with CEUS by the same examiner, and the intraplaque contrast enhancement was recorded. Raw images were stored in the scanner’s hard drive for offline analysis. Routine carotid ultrasonography and CEUS examination were performed as previously described (Cui et al., 2021).

### Intraplaque Neovascularization Analysis

**FIGURE 2:**
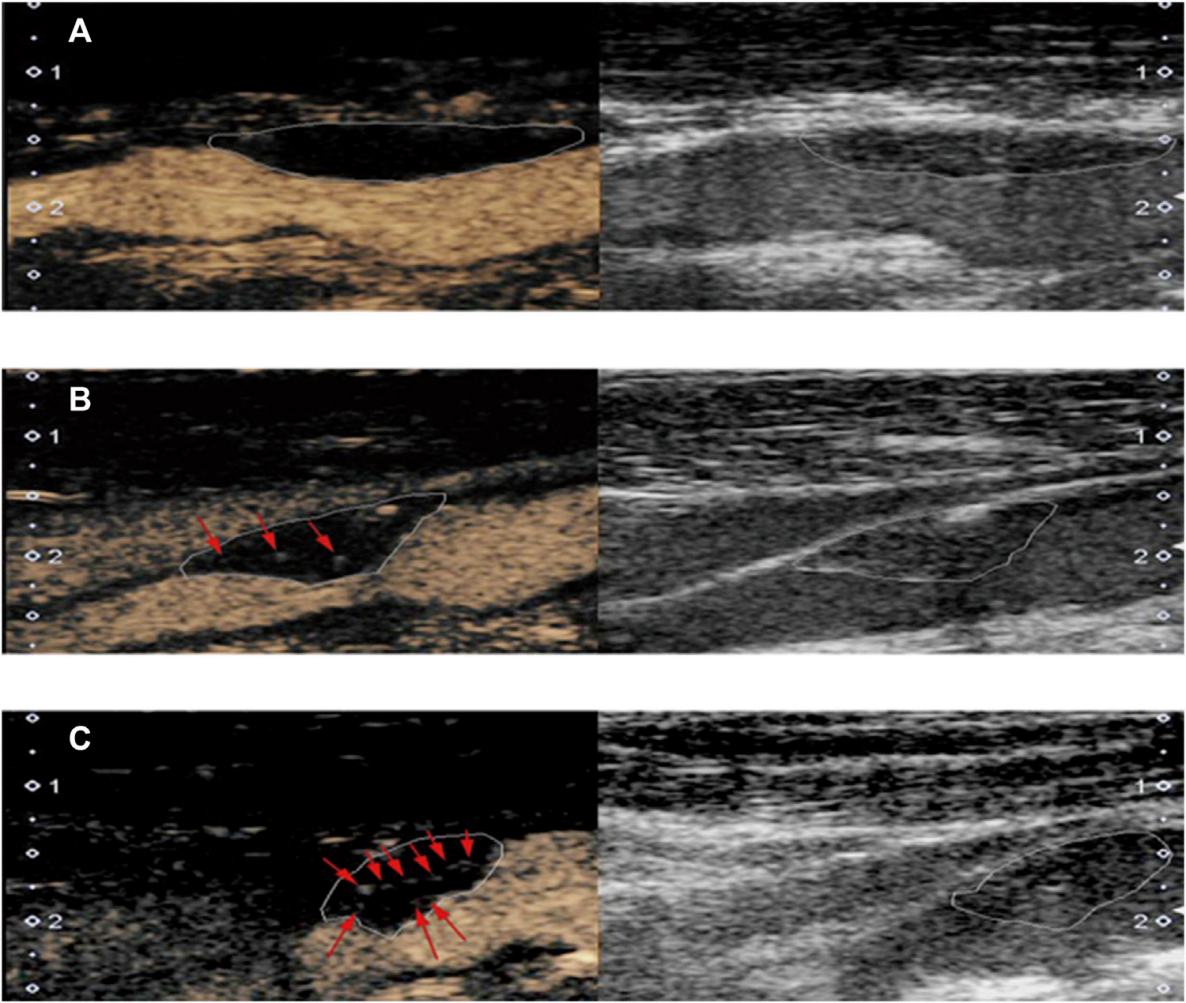
Typical examples of plaque based on contrast-enhanced ultrasonography (left) and carotid ultrasound (right). **(A)** grade 0: no enhancing microbubbles within the plaque; **(B)** grade 1: moderate enhancing microbubbles on the shoulder and/or adventitial side of the plaque; **(C)** grade 2: extensive enhancing microbubbles throughout the plaque.

Plaque neovascularization was classified into three levels (Mantella et al., 2019): grade 0, with no enhancing microbubbles within the plaque; grade 1, defined by moderate enhancing microbubbles confined to the shoulder and/or adventitial side of the plaque; and grade 2, defined by extensive enhancing microbubbles confined to the core of the plaque (Figure 2). Offline analysis of the raw images was performed by two sonographer experienced in CEUS (XYQ and WLJ, each with 10 years of experience) blinded to the patients’ clinical information and the results of the other analyses. In the case of discordant results, they were resolved jointly after discussion by both examiners.

### Clinical Data Collection and Follow-Up

We collected the following clinical variables: 1) demographic data including age and sex; 2) vascular event risk factors including history of smoking, alcohol consumption, diabetes mellitus, and hypertension; and 3) serological parameters including triglycerides, cholesterol (including low-density lipoprotein and high-density lipoprotein levels), fasting blood glucose; 4) carotid revascularization performed during follow-up; and 5) medication adherence.

In each patient, follow-up was performed every 6 months where the patient’s vascular events were recorded. The endpoint events were defined as ischemic stroke or other cardiovascular events. Ischemic stroke was defined as a sudden neurological deficit of more than 24 h confirmed by cranial computed tomography or magnetic resonance imaging. Cardiovascular events included non-fatal myocardial infarction, refractory unstable angina, unplanned coronary revascularization, or cardiac death. Patients taking medications for more than 80% of the days in the study period were considered to have good medication adherence.

### Statistical Analysis

We used the Shapiro-Wilk test to assess the normality of the data with *p* > 0.05 as the significance level for normal distribution. Normally distributed continuous variables are expressed as the mean ± standard deviation and non-normally distributed continuous variables are presented as medians and interquartile ranges. Frequencies and percentages (%) were used for categorical variables. The parameters were compared between the two groups using the Student’s t-test, the non-parametric rank-sum test, chi-square test, or Fisher’s exact test, as appropriate. The Kaplan-Meier method was used to plot survival curves, and the log-rank test was used to compare differences in endpoint event probabilities according to asymptomatic carotid stenosis with (grade 2) or without (grades 0 or 1) IPN. Finally, univariate and multivariate Cox proportional hazards regression analyses were used to determine the possible predictors of future vascular events in patients with asymptomatic carotid stenosis. Variables that were statistically significant in the univariate analysis were included in the multivariate analysis, and the final Cox model was defined. The level of significance was set at *p* < 0.05. Inter- and intra-observer agreements were assessed using Cohen’s kappa coefficient. Statistical analysis was performed using SPSS version 26.0 (IBM Corp, Armonk, NY, United States).

## Results

### Clinical Outcomes

After the inclusion and exclusion criteria were applied, we collected data from 50 participants with a mean age of 63 ± 9.8 years and 46 males (92%). During the follow-up period, 38 participants were “without events,” and 12 participants were “with events”. In the “with events” group, we observed isolated ischemic stroke in three participants (25%), isolated cardiovascular events in eight participants (66.7%), and both diseases in one participant (8.3%). Of the 19 participants who underwent revascularization, there were 14 participants in the “without events” group and 5 in the “with events” group.

### Clinical Characteristics

The clinical characteristics of the two groups are shown in Table 1. There was no statistical difference in age or sex between the two groups. The number of participants who smoked was significantly higher in the “with events” group than in the “without events” group (*p* = .019). Compared to the “without events” group, the “with events” group had higher levels of fasting blood glucose levels (*p* = .011) and a higher rate of diabetes mellitus (*p* = .014). However, there were no significant clinical differences in terms of other risk factors or carotid revascularization between the groups (*p* > .05).

**TABLE 1:** Clinical data of participants at baseline

	Total (*N* = 50)	without events (*n* = 38)	with events (*n* = 12)	*p*-value
Demographics				
Age (years)	63 (9.8)	62 (10.1)	67 (7.7)	0.113
Sex (men)	46 (92.0)	35 (92.1)	11 (91.7)	1.000
Risk factors				
Smoking	27 (54.0)	17 (44.7)	10 (83.3)	0.019
Alcohol abuse	23 (46.0)	17 (44.7)	6 (50.0)	0.750
Diabetes mellitus	11 (22.0)	5 (13.2)	6 (50.0)	0.014
Hypertension	31 (62.0)	25(65.8)	6 (50.0)	0.496
Family history	21 (42.0)	14 (36.8)	7 (58.3)	0.189
Serological indicators				
Triglyceride level (mmol/L)	1.51 (1.05–1.70)	1.51 (1.05–2.04)	1.50 (1.01–1.65)	0.547
Cholesterol level (mmol/L)	3.93 (3.35–4.75)	3.91 (3.35–4.78)	4.13 (3.44–4.67)	0.691
LDL level (mmol/L)	2.46 (1.87–3.06)	2.36 (1.74–2.93)	2.71 (2.22–3.49)	0.195
HDL level (mmol/L)	1.12 (0.91–1.24)	1.12 (0.92–1.25)	1.11 (0.87–1.24)	0.586
FBG level (mmol/L)	5.08 (4.67–5.98)	4.88 (4.60–5.56)	5.76 (5.33–6.85)	0.011
Carotid revascularization	19 (38.0)	14 (36.8)	5 (41.7)	1.000
Medication adherence	41 (82.0)	32 (84.2)	9 (75.0)	0.668

HDL, high-density lipoprotein; LDL, low-density lipoprotein; FBG, fasting blood-glucose.

Numbers are presented as n (%), mean (SD) or median (IQR).

### Ultrasonography Characteristics of Carotid Plaques in Participants

There were more participants with diffuse IPN (grade 2) in the “with events” group than in the “without events” group (*p* = .027) and the proportion of hypoechoic plaques (types I and II) was greater in participants in the “with events” group compared with the “without events” group (*p* = .041). There was no significant difference in the degree of carotid stenosis between the two groups (*p* = .750) (Table 2).

**TABLE 2:** Plaque characteristics of participants

	Total (*N* = 50)	without events (*n* = 38)	with events (*n* = 12)	*p*-value
Plaque echogenicity				0.041
Types Ⅰ and Ⅱ	29 (58.0)	19 (50.0)	10 (83.3)	
Types Ⅲ and Ⅳ	21 (42.0)	19 (50.0)	2 (16.7)	
CEUS				0.027
Grades 0 and 1	35 (70.0)	30 (78.9)	5 (41.7)	
Grade 2	15 (30.0)	8 (21.1)	7 (58.3)	
Degree of carotid stenosis				0.750
Mild and moderate stenosis	27 (54.0)	21 (55.3)	6 (50.0)	
Severe stenosis	23 (46.0)	17 (44.7)	6 (50.0)	

CEUS, contrast-enhanced ultrasound.

Numbers are presented as *n* (%).

### Risk Factors of Intraplaque Neovascularization

According to the presence of neovascularization in the carotid plaque, participants were divided into “absence of neovascularization” (grade 0, *n* = 13 participants) and “presence of neovascularization” (grades 1 and 2, *n* = 37 participants) groups. There was a significant difference between the two groups in terms of sex—there were more men in the “presence of neovascularization” group than in the ‘absence of neovascularization’ group (*p* = .003)—but no significant difference in age (*p* = .185). Compared with the “absence of neovascularization” group, there were more smokers in the “presence of neovascularization” group (*p* = .009). There were no significant differences in other risk factors for vascular events between the two groups (*p* > .05) (Table 3). For the assessment of CEUS images, the agreement for intra-observer reliability was 0.781 and 0.753 for inter-reviewer reliability, both of which correspond to good reliability.

**TABLE 3:** Relationship between IPN and convention risk factors

	Absence of neovascularization (Grade 0)	Presence of neovascularization (Grades 1 and 2)	*p*-value
	(*n* = 13)	(*n* = 37)	
Demographics	60 (9.92)	64 (9.59)	0.185
Age (years)			
Sex (men)	9 (69.2)	37 (100.0)	**0.003**
Risk factors			
Smoking	3 (23.1)	24 (64.9)	**0.009**
Alcohol abuse	4 (30.8)	19 (51.4)	0.200
Diabetes mellitus	3 (23.1)	9 (21.6)	1.000
Hypertension	10 (76.9)	21 (56.8)	0.320
Serological indicators			
Triglyceride level (mmol/L)	1.56 (1.10–1.68)	1.47 (1.05–1.86)	0.465
Cholesterol level (mmol/L)	3.90 (3.51–4.66)	4.03 (3.33–4.76)	0.391
LDL level (mmol/L)	2.31 (1.94–2.89)	2.48 (1.74–3.15)	0.518
HDL level (mmol/L)	1.13 (1.07–1.25)	1.09 (0.90–1.25)	0.751
FBG level (mmol/L)	4.89 (4.64–6.34)	5.22 (4.72–5.85)	0.532

HDL, high-density lipoprotein; LDL, low-density lipoprotein; FBG, fasting blood-glucose.

Numbers are presented as *n* (%), mean (SD) or median (IQR).

### Predictors of Endpoint Events

**FIGURE 3:**
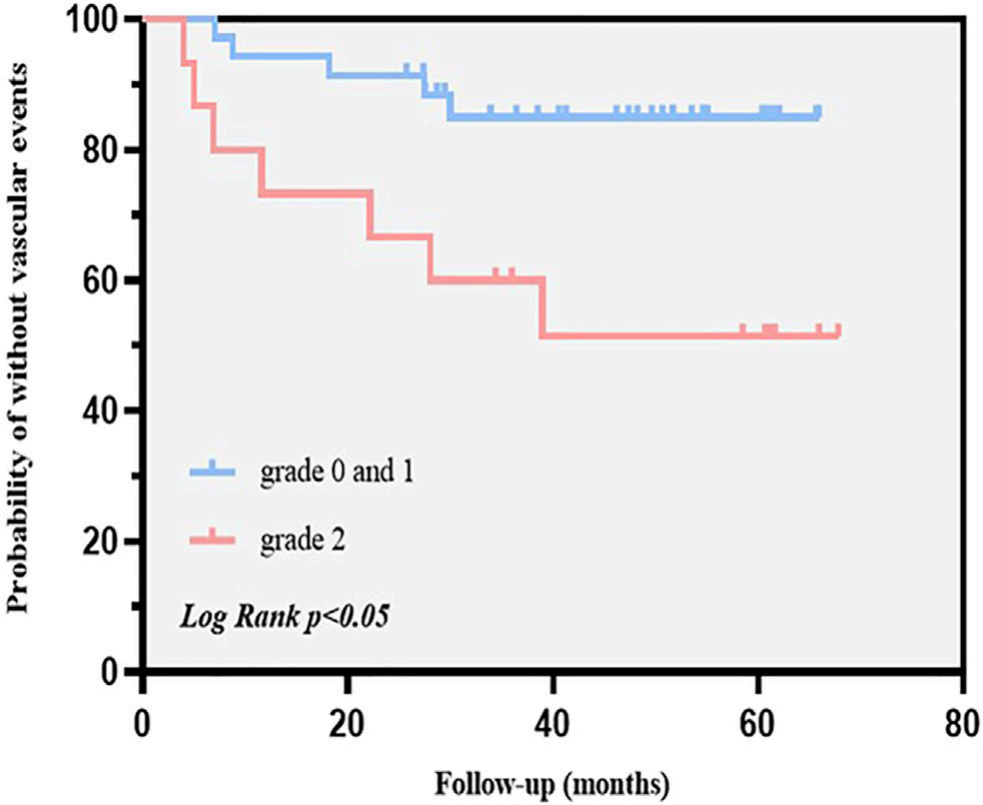
Kaplan-Meier survival curves show endpoint event probabilities according to the contrast-enhanced plaque pattern. Patients with asymptomatic carotid stenosis and intraplaque neovascularization (IPN) (grade 2) have a significantly higher probability of future vascular events than in those with IPN (grade 0 and 1). Censored data are indicated with vertical bars.

To investigate potential confounding factors and assess whether IPN is an independent risk factor for future vascular events in patients with asymptomatic carotid stenosis, Kaplan-Meier survival curves were plotted (Figure 3) and univariate and multivariate Cox regression models were fitted (Table 4). The Kaplan-Meier survival curves showed that grade 2 IPN assessed using CEUS was associated with a significantly higher probability of future vascular events in asymptomatic carotid stenosis patients. The parameters (smoking, diabetes mellitus, grade 2 IPN) related to primary outcomes (*p* < .05) were included in the multivariate analysis to obtain the final prediction model. In the multivariable Cox proportional-hazards models, the adjusted hazard ratios (HRs) for grade 2 IPN were 4.530 (95% CI, 1.337–15.343) and the adjusted HR for diabetes mellitus was 5.744 (95% CI, 1.694–19.478). Smoking was also included, although the HR included 1 in the CI, with an adjusted HR of 4.159 (95% CI, 0.892–19.386) (*p* = .070).

**TABLE 4:** Predictors of primary outcomes

	Univariate analysis		Multivariate analysis	
	HR (95% CI)	*p*-value	HR (95% CI)	*p*-value
Age (years)	1.053 (0.987–1.123)	0.120		
Sex (men)	0.869 (0.112–6.736)	0.892		
Smoking	5.160 (1.128–23.614)	0.034	4.159 (0.892–19.386)	0.070
Alcohol abuse	1.340 (0.432–4.159)	0.612		
Diabetes mellitus	4.886 (1.566–15.245)	0.006	5.744 (1.694–19.748)	0.005
Hypertension	0.556 (0.179–1.729)	0.310		
Family history	1.734 (0.550–5.468)	0.348		
Triglyceridelevel (mmol/L)	0.601 (0.213–1.694)	0.336		
Cholesterol level (mmol/L)	0.966 (0.591–1.578)	0.891		
LDL level (mmol/L)	1.257 (0.757–2.087)	0.376		
HDL level (mmol/L)	0.373 (0.046–3.041)	0.357		
FBG level (mmol/L)	1.196 (0.905–1.581)	0.209		
Carotid revascularization	1.249 (0.396–3.949)	0.705		
Medication adherence	0.604 (0.163–2.243)	0.452		
Hypoechoic plaque	4.116 (0.901–18.809)	0.068		
IPN (grade 2)	3.914 (1.241–12.342)	0.020	4.530(1.337–15.343)	0.015
Severe carotid stenosis (≥70%)	1.221 (0.393–3.788)	0.730		

IPN, intraplaque neovascularization; CI, confidence interval; HR, hazard ratio.

## Discussion

This study aimed to explore the association between IPN assessed using CEUS and the occurrence of future ischemic events in asymptomatic carotid stenosis patients. Our results indicate that carotid IPN (grade 2) detected by CEUS is an independent predictor of future vascular events in asymptomatic carotid stenosis patients after adjustment for traditional vascular risk factors. This implies that CEUS may be an attractive risk stratification method for future ischemic events in asymptomatic carotid stenosis patients, allowing for the identification of high-risk subgroups. In addition, our results confirm that atherosclerosis is a systemic and diffuse disease that may occur in both the coronary and cerebral arteries.

During the study period, ischemic stroke (*n* = 3), cardiovascular events (*n* = 8), and both ischemic stroke and cardiovascular events (*n* = 1), were reported. We did not expect that there would be more cardiovascular events compared to others due to several factors. Firstly, some patients underwent carotid revascularization during follow-up. Previous studies have shown that revascularization in patients with asymptomatic severe carotid stenosis can reduce the incidence of future cerebrovascular events but cannot reduce the risk of cardiovascular disease (Michael et al., 1995; Chatzikonstantinou et al., 2012). Secondly, it is reported that many patients with asymptomatic carotid stenosis have concurrent coronary artery disease (CAD) (Sulženko et al., 2019). A study of Japanese patients found that perioperative screening for CAD showed that patients planning to receive carotid artery stenting often had silent CAD (Sulženko et al., 2019). Silent myocardial infarction, which cannot be detected by echocardiogram, is often detected by contrast-enhanced magnetic resonance angiography in patients with carotid stenosis, illustrating from another perspective the systemic nature of atherosclerosis (Quarto et al., 2015).

It has been suggested that patients with asymptomatic carotid stenosis should be assessed for high-risk plaques rather than just the degree of stenosis. Identifying patients at high risk for vascular events could help in clinical decision-making regarding appropriate drug treatment or the use of more invasive strategies (Kamtchum-Tatuene et al., 2020). Further evidence from a study using analytic models to predict cost and quality-adjusted life-years for stroke prevention showed that patients who underwent revascularization based on carotid stenosis progression or ultrasound plaque echogenicity had the fewest stroke events and the longest life years (Baradaran et al., 2019). In our study, the echogenicity of plaques was not entered into the final model. However, in Fisher’s exact test, the carotid hypoechoic plaques (types I and II) were statistically different between the two groups, which may be related to the small sample size. Previous studies reported that the echogenicity of the plaques was negatively correlated with the plaque neovascularization grades. Therefore, CEUS examination should be performed in patients with carotid plaques that are hypoechoic (types I and II) (Staub et al., 2011; Li et al., 2019). Thus, assessing plaque vulnerability is a cost-effective risk stratification method in patients with asymptomatic carotid stenosis.

As reported in the literature, in patients with symptomatic carotid stenosis, IPN, as one of the markers of plaque vulnerability, is an independent risk factor not only for cerebrovascular events but also for coronary atherosclerotic disease. For example, histological studies indicate that plaques with high-contrast enhancement grades showed more neovascularization histologically (Coli et al., 2008). Retrospective studies have illustrated that the degree of intraplaque enhancement detected by CEUS is higher in patients who had experienced a transient ischemic attack/ischemic stroke than in those without a history of ischemic events (Xiong et al., 2009). Furthermore, intraplaque neovessels are more easily detected in patients with a history of cardiovascular disease than in those without (Staub et al., 2010). Recent prospective studies have found that IPN detected by CEUS is an independent predictor of ischemic stroke recurrence (Camps-Renom et al., 2020). One study suggests that IPN predicts complex and significant coronary heart disease (CHD) and stratifies risk for future cardiovascular events (Zhu et al., 2013). Thus, IPN is more prevalent in patients with a history of ischemic cardio- and cerebrovascular disease and is associated with an increased risk of future ischemic stroke and CHD.

However, few studies have reported the predictive value of IPN for vascular events in patients with asymptomatic carotid stenosis. In our study, the association between IPN and future vascular events in patients with asymptomatic carotid stenosis was prospectively explored. We demonstrated that intraplaque neovascularization detected by CEUS remained an independent predictor of future vascular events after adjusting for confounders in the Cox regression multivariate analysis, similar to the results of recent studies. For example, a prospective observational study of 818 patients who had undergone carotid endarterectomy found that 196 (24%) patients had vascular events during a mean follow-up of 2.3 years, and histological analysis of plaques showed that intraplaque neovessel density was an independent predictor of the occurrence of future vascular events (Hellings et al., 2010). A meta-analysis of 20 ,751 participants suggests that the prevalence of high-risk features was not associated with the degree of stenosis and that patients with high-risk plaques, with or without focusing on severe stenosis, had a higher rate of ipsilateral ischemic cerebrovascular events than those without high-risk plaques (Kamtchum-Tatuene et al., 2020).

In the present study, smoking was strongly associated with future vascular events and intraplaque neovascularization in patients with asymptomatic carotid stenosis. Diabetes mellitus is independently associated with future vascular events in patients with asymptomatic carotid stenosis. Smoking and diabetes mellitus are well-known risk factors for vascular events. Cigarette smoke not only promotes oxidative stress by directly accelerating vascular injury and activating inflammatory cells but is also related to endothelial dysfunction (Shah and Cole, 2010). Animal studies have shown that IPN is associated with disturbed vascular endothelial growth factor signaling pathways and nitric oxide signaling. Many studies have found that patients with diabetes are in a prothrombotic and procoagulant state. Circulating inflammatory signals initiated by aldose reductase activate glucotoxicity and the release of local inflammatory chemoattractants worsens the thrombotic state. Recent literature has shown that diabetes and obesity cause microvascular dysfunction by inducing a pro-inflammatory state (Gallinoro et al., 2021). Moreover, hyperglycemia is associated with oxidative stress (Rodriguez-Araujo et al., 2018). Therefore, these factors appear to ultimately promote vulnerable plaque formation by inducing oxidative stress, endothelial dysfunction, vascular inflammation, and hypercoagulability. These findings emphasize the importance of controlling risk factors in the management of ischemic events.

Our study has some limitations. First, the sample size is small, and it was a single-center study. A multicenter, large-sample study is needed to verify our conclusions. Second, there was male predominance among the study participants, and therefore, it was not possible to investigate whether sex was a risk factor for future vascular events. Finally, we selected only the thickest plaque in each patient, which may have led to a potential spectrum bias.

In conclusion, our findings demonstrate that grade 2 IPN detected by CEUS is an independent predictor of future vascular events in patients with asymptomatic carotid stenosis. Plaque vulnerability often coexists in the systemic vascular bed, and attention should be paid to vulnerable plaques in asymptomatic patients with carotid stenosis.

## Data Availability

The raw data supporting the conclusion of this article will be made available by the authors, without undue reservation.
